# Genome-wide search reveals a novel GacA-regulated small RNA in *Pseudomonas *species

**DOI:** 10.1186/1471-2164-9-167

**Published:** 2008-04-13

**Authors:** Nicolas González, Stephan Heeb, Claudio Valverde, Elisabeth Kay, Cornelia Reimmann, Thomas Junier, Dieter Haas

**Affiliations:** 1 Département de Microbiologie Fondamentale, Biophore, Université de Lausanne, CH-1015 Lausanne, Switzerland; 2 Institute of Infection, Immunity and Inflammation, Center for Biomolecular Sciences, University of Nottingham, Nottingham NG7 2RD, UK; 3 Department of Genetic Medicine and Development, University of Geneva, Rue Michel-Servet 1, CH-1211 Geneva, Switzerland; 4 Departamento de Ciencia y Tecnología, Programa Interacciones Biológicas, Universidad Nacional de Quilmes, Saenz Peña 352, Bernal B1876BXD, Argentina; 5 UMR5163/CNRS-UJF, Institut Jean-Roget, Grenoble, France

## Abstract

**Background:**

Small RNAs (sRNAs) are widespread among bacteria and have diverse regulatory roles. Most of these sRNAs have been discovered by a combination of computational and experimental methods. In *Pseudomonas aeruginosa*, a ubiquitous Gram-negative bacterium and opportunistic human pathogen, the GacS/GacA two-component system positively controls the transcription of two sRNAs (RsmY, RsmZ), which are crucial for the expression of genes involved in virulence. In the biocontrol bacterium *Pseudomonas fluorescens *CHA0, three GacA-controlled sRNAs (RsmX, RsmY, RsmZ) regulate the response to oxidative stress and the expression of extracellular products including biocontrol factors. RsmX, RsmY and RsmZ contain multiple unpaired GGA motifs and control the expression of target mRNAs at the translational level, by sequestration of translational repressor proteins of the RsmA family.

**Results:**

A combined computational and experimental approach enabled us to identify 14 intergenic regions encoding sRNAs in *P. aeruginosa*. Eight of these regions encode newly identified sRNAs. The intergenic region 1698 was found to specify a novel GacA-controlled sRNA termed RgsA. GacA regulation appeared to be indirect. In *P. fluorescens *CHA0, an RgsA homolog was also expressed under positive GacA control. This 120-nt sRNA contained a single GGA motif and, unlike RsmX, RsmY and RsmZ, was unable to derepress translation of the *hcnA *gene (involved in the biosynthesis of the biocontrol factor hydrogen cyanide), but contributed to the bacterium's resistance to hydrogen peroxide. In both *P. aeruginosa *and *P. fluorescens *the stress sigma factor RpoS was essential for RgsA expression.

**Conclusion:**

The discovery of an additional sRNA expressed under GacA control in two *Pseudomonas *species highlights the complexity of this global regulatory system and suggests that the mode of action of GacA control may be more elaborate than previously suspected. Our results also confirm that several GGA motifs are required in an sRNA for sequestration of the RsmA protein.

## Background

In bacteria, > 150 non-coding small RNAs (sRNAs) have been described [1]. The first bacterial sRNAs were discovered in *Escherichia coli*, either fortuitously due to their abundance or by the observation of phenotypes conferred by their overexpression. Some abundant and stable sRNAs were found early in gel electrophoretic analysis. They include 4.5 S RNA, which is implicated in protein export; a component of RNase P, which participates in tRNA processing; and tmRNA, which has an important role in translational quality control [2]. The majority of sRNAs was identified by systematic approaches, principally in *E. coli*. Computational searches mainly focused on intergenic regions (IgRs) and were combined with predictions of promoters and of rho-independent transcription terminators [3,4]. The QRNA algorithm, which takes into account the sequence homology of IgRs in closely related species as well as conserved secondary structures [5], was combined with experimental methods to identify several sRNAs, e.g. those involved in sporulation of *Bacillus subtilis *[6].

Most bacterial sRNAs studied have regulatory roles in gene expression, occurring in many instances at a post-transcriptional level. In one type of post-transcriptional regulation, which is usually governed by the RNA chaperone Hfq in Gram-negative bacteria, sRNAs interact with specific mRNA targets, thereby modifying the accessibility of the Shine-Dalgarno sequence to the translational machinery and often altering the stability of the mRNA. For instance, in *E. coli*, the iron-containing superoxide dismutase SodB is regulated by iron availability via the sRNA RyhB, which base-pairs with *sodB *mRNA. This interaction blocks ribosome access and favors nucleolytic degradation of the mRNA [7]. A second type of post-transcriptionally active sRNAs interacts with RNA-binding regulatory proteins of the RsmA/CsrA family. RsmA (regulator of secondary metabolism) and CsrA (carbon storage regulator) can act as translational repressors; sRNAs having high affinity for these proteins are therefore able to relieve translational repression by sequestering them [8].

In pseudomonads, few sRNAs have been studied. In *P. fluorescens*, the sRNAs RsmX, RsmY and RsmZ were detected by their binding capacity to the regulatory protein RsmA [9,10], by their sequence similarity with already identified sRNAs [11], or by multicopy suppression of a *gacA *mutation [12]. The GacS/GacA two-component system (which is homologous to BarA/UvrY in *E. coli*) activates the transcription of these three sRNAs. When they are present in high concentrations, they titrate the RNA-binding proteins RsmA and its homolog RsmE, resulting in enhanced translational expression of genes involved in biocontrol of plant root diseases and in resistance to oxidative stress [10,13,14]. Biocontrol factors of strain CHA0 are secondary metabolites (e. g., hydrogen cyanide [HCN]) and lytic exoenzymes [15]. In *P. aeruginosa*, the Gac/Rsm system involves two sRNAs, RsmY and RsmZ [16]. As part of the quorum sensing machinery this regulatory system not only controls the expression of genes specifying exoproducts such as HCN, pyocyanin and elastase, but also upregulates the expression of the *rhlI *gene, which codes for the enzyme synthesizing the quorum sensing signal *N*-butanoyl-homoserine lactone [17,18]. Furthermore, a search for Fur-box motifs in IgRs of *P. aeruginosa *led to the discovery of two iron-regulated sRNAs, PrrF1 and PrrF2 [19]. More recently, a study using a program that combines several predictive features mentioned above revealed a total of 17 sRNAs in strain PAO1 [20].

We have begun to search for new GacA-regulated sRNAs in *Pseudomonas *spp., by applying the QRNA method to IgRs of various *Pseudomonas *spp., combined with a prediction of rho-independent terminators and, where appropriate, putative promoters. Eight sRNAs were newly identified in *P. aeruginosa *by Northern blotting experiments. By comparing sRNA expression in the wild-type with that in a *gacA *mutant, we discovered a novel GacA-controlled sRNA termed RgsA. We analyzed its regulation and involvement in biocontrol factor expression and oxidative stress response in *P. fluorescens *CHA0. Moreover, we show that RgsA expression in *P. aeruginosa *and *P. fluorescens *strictly depends on the stress sigma factor RpoS.

## Results

### Prediction and screening of sRNAs in *P. aeruginosa *PAO1

The majority of the bacterial sRNA genes found to date is located in IgRs [3,21] and the size of the sRNAs generally varies between 50 and 400 nt [1,22]. Accordingly, we based our search for sRNAs in *P. aeruginosa *PAO1 on the criterion that sRNA genes should be located in IgRs that are larger than 50 bp. A total of 3168 IgRs fulfilled this condition. We then assumed that functional sRNA sequences should be conserved in closely related species [4,20,23] and we used BLASTn to search for sequences that are homologous to the 3168 IgRs of *P. aeruginosa *in the genomic sequences of five related pseudomonads (*Pseudomonas putida *KT2440, *Pseudomonas syringae *pv. *tomato *DC3000, *Pseudomonas fluorescens *Pf0-1, *P. fluorescens *SBW25 and *P. fluorescens *Pf-5). Only those PAO1 IgRs were selected that shared more than 65% sequence identity with a genomic sequence of at least one other pseudomonad. The alignments resulting from the BLAST program were then subjected to QRNA analysis [5]. In pairwise alignments of homologous sequences, this algorithm evaluates pairs of nucleotide substitutions and calculates their probabilities of having structural or codon usage conservation and discriminates sequence pairs without a sufficient number of substitutions. RNAs having substitutions with a bias towards codon usage are likely to code for polypeptides whereas pairs of substitutions that maintain stem-loop structures have a higher probability to reflect secondary structure conservation of non-coding RNAs. Although the sequences subjected to BLASTn analysis consisted only of intergenic, non-coding regions according to the *P. aeruginosa *PAO1 genome annotations, sequences whose coding probabilities calculated by QRNA were found to be high were nevertheless retained for further analysis. By applying QRNA in this way, 162 out of the initial 3168 IgRs from *P. aeruginosa *PAO1 were retained (see Table in Additional file 1). Among these 162 IgRs, 32 were found to contain exclusively tRNA or rRNA genes and were not considered further. In the 130 remaining regions, the presence of rho-independent terminators was assessed using TransTerm [24] set at a confidence cut-off of 93% (i.e., expecting 93% of all predictions to be correct).

### Experimental observation of sRNAs in *P. aeruginosa *PAO1

**Figure 1:**
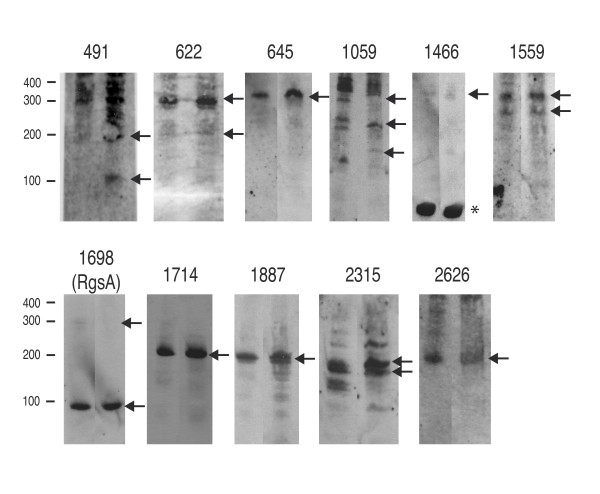
**Detection of sRNAs in *P. aeruginosa *by Northern blots**. Total RNA was purified from cultures of *P. aeruginosa *strain PAO1 grown in NYB until exponential phase (first lane in each blot) and stationary phase (second lane in each blot). Experimental conditions are described in Methods. The blots for the annotated sRNA genes encoding 4.5S RNA (in IgR 888), RNase P RNA (in IgR 2510) and PrrF1, 2 (in IgR 2667) are not shown. The approximate positions of RNA standards are shown on the left. The main transcripts are pointed out by arrows. A tRNA^Cys ^gene is embedded in the IgR 1466; its transcript is indicated by an asterisk.

IgRs occurring in *P. aeruginosa *and at least two other pseudomonads and having recognizable promoter elements (e.g., for the housekeeping sigma factor RpoD or the stress sigma factor RpoS) were preferentially chosen for Northern blot analysis. Cultures of *P. aeruginosa *PAO1 were grown in nutrient yeast broth (NYB) to exponential or stationary phase and total RNA was extracted. Northern blot analysis was carried out with digoxigenin (DIG)-labeled probes, each covering an entire IgR. Among 49 IgRs thus analyzed, 14 were reproducibly found to express sRNAs (Table 1). Figure 1 shows all transcripts revealed, except for four sRNAs that had been described previously: 4.5S RNA (encoded by the *ffs *gene in IgR 888) [2,25], an RNase P component found in many bacteria (encoded by *rnpB *in IgR 2510) [2,20], and PrrF1 and PrrF2 found in *P. aeruginosa *(encoded by *prrF1 *and *prrF2 *in IgR 2667) [19]. They were considered to validate the method and not investigated further. Three additional sRNAs (encoded by IgRs 491, 1698, 1887; Table 1) are among the 17 *P. aeruginosa *sRNAs described by Livny *et al. *[20]. In that study, the three sRNAs are designated P5, P16 and P20, respectively. In conclusion, we detected eight new sRNAs encoded by the remaining IgRs.

**Table 1:** sRNAs transcripts observed in *P. aeruginosa *PAO1

Intergenic region ^a^	Coordinates in PAO1 genome (bp)	5' gene		3' gene		sRNA		
				
		Length (bp)	Dir.^b^	Length (bp)	Dir.^b^	Predicted length (nt)^c^	Observed length (nt)^d^	Dir.^g^
**491**	912780–913085	1185	<	486	>	90	100, 200	>
622	1140860–1141267	1146	>	465	>		200, 300	?
645	1204782–1205770	1149	>	1767	>	300 ± 10	300	<
888 = *ffs*	1668833–1669085	378	>	867	<	113	90	>
1059	1996807–1997508	1659	<	1041	<		150, 200, 300	?
1466	2918212–2918965	1041	>	534	<		300	?
1559	3112151–3112876	519	>	900	>		250, 300^e^	?
**1698 ***= rgsA*	3318657–3318881	1134	>	777	<	120 ± 10	100, 300	>
1714	3360654–3360873	1386	<	3474	>		200	?
**1887**	3705161–3705888	1995	<	603	<	210 ± 10	200	<
2315	4536493–4536919	660	<	1122	<	160 ± 10	180	<
2510 = *rnpB*	4956029–4956732	452	<	849	<	208	200	<
2626	5196833–5197184	1464	>	576	>		200	>
2667 = *prrF1,2*	5283906–5284368	798	>	888	<	110, 110	100, 110 ^f^	>>

^a ^Transcripts that were also found by Livny *et al. *[20] are shown in boldface

^b ^Dir., direction of flanking genes

^c ^Lengths of transcripts described in other studies are indicated by their experimental values; otherwise, predicted lengths are estimates ± 10 nt

^d ^Bands that were observed in cells grown in NYB, GGP or MME medium; estimated size ± 10 nt

^e ^Not tested in GGP and MME medium

^f ^No transcript was observed in cells grown in NYB or GGP in exponential phase; transcripts were present in stationary phase cells grown in all media

^g ^Dir., direction of sRNA genes. When rho-independent terminators are predicted to be present, the direction of the sRNA is annotated (> or <). When the direction is unknown, this is indicated by a question mark

All transcripts were also detected when cells had been cultivated in the iron-limited medium GGP or in the minimal medium MME (data not shown), except for 1559 sRNA, which was not tested in these alternative media. Six regions (IgRs 491, 622, 1059, 1559, 1698, 2315) produced more than one transcript (Figure 1); we only indicate the most prevalent bands in this figure. Multiple transcripts can be due to processed sRNAs, 5' leader mRNA sequences or 3' mRNA fragments related to flanking genes [26]. Three other regions (IgRs 1714, 1887, 2626) each revealed a major band, together with possible degradation products. Whenever the sRNA transcript length could be predicted with reasonable certainty (Table 1), at least one of the main transcripts observed had the expected size (Figure 1). IgR 491 specified a transcript that had previously been observed [20]; we deduce from the sequence that it originates from an RpoS-dependent promoter. The 180-nt sRNA encoded by IgR 2315, which is located between the *ribC *and *ribD *genes, is a homolog of *sroG*, a transcript resulting from the cleavage of a riboswitch element found upstream of *ribB *in *E. coli *[27]. The *P. aeruginosa sroG *homolog was slightly longer than the *sroG *transcript in *E. coli *(147 nt) [27]. The 2315 sRNA of *P. aeruginosa *may be involved in regulation of riboflavin biosynthesis, by analogy with the homologous element in *E. coli *[28,29]. In four cases (IgRs 645, 1698, 1887, and 2315), the coordinates of the sRNA genes can be deduced either from recognizable promoter and terminator elements or by sequence comparison with an *E. coli *RNA (Additional file 2 for the IgRs).

### GacA- and RpoS-regulated expression of the 1698 sRNA in *P. aeruginosa *PAO1 and *P. fluorescens *CHA0

**Figure 2:**
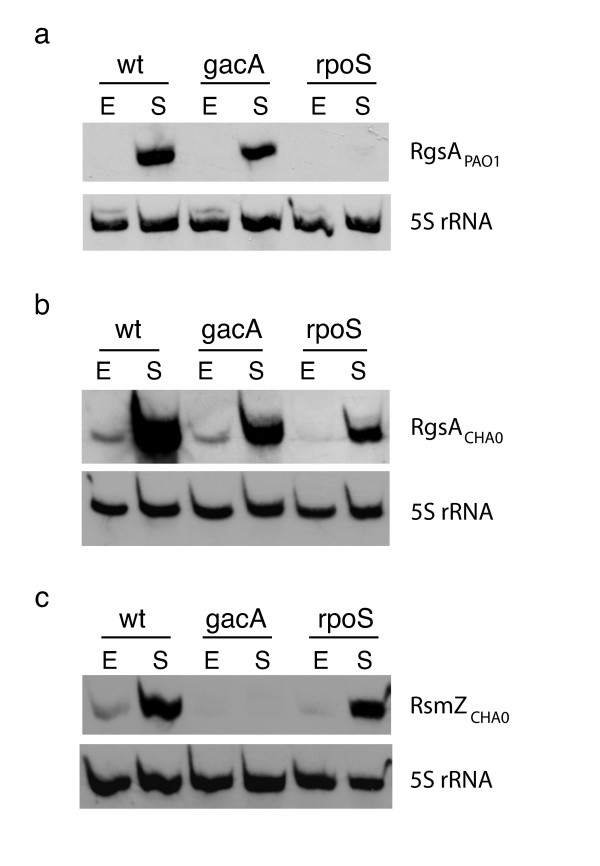
**Detection of transcriptional control of RgsA (1698) sRNA in *P. aeruginosa *and in *P. fluorescens *by Northern blot**. **a**. Hybridization of cross-linked total RNA from *P. aeruginosa *PAO1 (wt), PAO6281 (*gacA *mutant) and PAO1-rpoS (*rpoS *mutant) with a double-stranded DNA probe prepared with primers 1698F (PAO1) and 1698R (PAO1) (additional file 3). RNA preparations were obtained from PAO1 (wild-type) in exponential phase (OD_600 _≅ 0.8) and stationary phase (OD_600 _≅ 4.9); from PAO6281 (*gacA*) in exponential phase (OD_600 _≅ 0.7) and stationary phase (OD_600 _≅ 4.8); from PAO1-rpoS (*rpoS*) in exponential phase (OD_600 _≅ 0.7) and stationary phase (OD_600 _≅ 5.0).**b**. Hybridization cross-linked total RNA from *P. fluorescens *CHA0 (wt), CHA89 (*gacA *mutant) and CHA815 (*rpoS *mutant) with a double-stranded DNA probe prepared with primers 1698F (CHA0) and 1698R (CHA0) (additional file 3). RNA was extracted from CHA0 (wild-type) in exponential phase (OD_600 _≅ 0.5) and stationary phase (OD_600 _≅ 6); CHA89 (*gacA*) in exponential phase (OD_600 _≅ 0.6) and stationary phase (OD_600 _≅ 6); CHA815 (*rpoS*) in exponential phase (OD_600 _≅ 0.6) and stationary phase (OD_600 _≅ 5.0). **c. **Hybridization of the same RNA preparations from *P. fluorescens *as in **b**, with an RsmZ probe synthesized with primers RsmZF (CHA0) and RsmZR (CHA0) (additional file 3). As a loading control, 5S rRNA was revealed in all samples with a probe synthesized with primers 5S rRNA-1 and 5S rRNA-2 (additional file 3). E: exponential phase, S: stationary phase.

The expression of the 11 sRNAs shown in Figure 1 was tested in the *P. aeruginosa gacA *mutant PAO6281 by Northern blotting. The 1698 sRNA was the only transcript that showed a significantly decreased expression in the *gacA *mutant, compared with that in the wild-type PAO1 (data not shown). This 120-nt transcript was further characterized. It was virtually absent from strain PAO1 during the exponential phase, but was produced abundantly in the stationary phase. In the *gacA *mutant, the expression of the 1698 sRNA was about two-fold lower than in the wild-type (Figure 2a), suggesting positive regulation by GacA. Moreover, in a mutant lacking the stress/stationary phase sigma factor RpoS, PAO1-rpoS [30], no expression of this RNA was detected (Figure 2a). We therefore named this sRNA RgsA (for regulation by GacA and stress).

**Figure 3:**
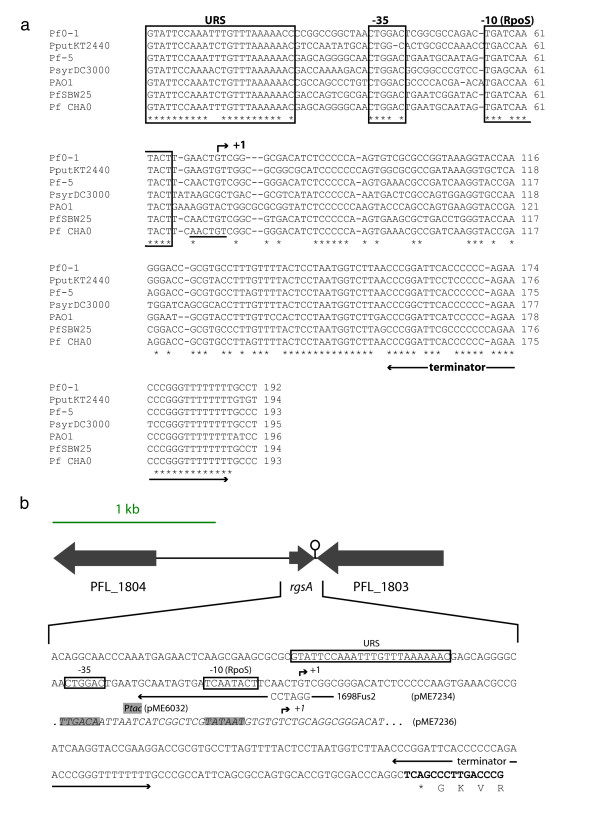
**Conservation of IgR 1698 in pseudomonads**. **a**. Alignment of the *P. aeruginosa *PAO1 IgR 1698 with homologous sequences present in *P. fluorescens *Pf0-1, *P. putida *KT2440, *P. fluorescens *Pf-5, *P. syringae *pv. *tomato *DC3000 and *P. fluorescens *SBW25. The IgR 1698 sequence of *P. fluorescens *CHA0, which was determined experimentally in this study, is added to the alignment. The alignment was made using ClustalW [53]. Conserved nucleotides are marked with an asterisk. +1, putative transcription start; URS, putative upstream regulating sequence; -35 and -10, postulated sigma-38 (RpoS) promoter elements. The sequence replaced by a BamHI site in the *rgsA*_*CHA0*_-*lacZ *fusion construct pME7234 is underlined. **b**. Organization of the 2.4-kb *rgsA *gene region in strain CHA0. The sequence of the downstream ORF PFL_1803 is shown in boldface with its translated sequence below. The 1698Fus2 primer employed to construct pME7234 is shown as an arrow, with the cloning BamHI site detailed. Part of the sequence of the *rsgA*_*CHA0 *_overexpressing construct pME7236 is shown in italics; the *tac *promoter (P*tac*) is indicated by grey boxes and its transcription start by *+1*.

The RgsA sRNA was also observed in *P. fluorescens *CHA0 (Figure 2b), a strain closely related to *P. fluorescens *Pf-5, whose genome has been sequenced. There is 99% identity between the two *P. fluorescens *strains for the IgR 1698 (Figure 3a). In strain CHA0, the band corresponding to this sRNA was faint in the wild-type CHA0, the *gacA *mutant CHA89 and the *rpoS *mutant CHA815 during the exponential phase. In the stationary phase, the transcript was observed clearly in the wild-type, whereas the expression was strongly reduced in the *gacA *mutant and in the *rpoS *mutant (Figure 2b), suggesting that again both GacA and RpoS contribute to the regulation of RgsA RNA.

As a control, we included the RsmZ sRNA of strain CHA0 in this Northern blot analysis. Confirming earlier results [9], we see that *rsmZ *expression depends on positive control by GacA, but is independent of RpoS (Figure 2c).

### Location of the *rgsA *gene in pseudomonads

The *rgsA *gene is strongly conserved in seven related pseudomonads (Figure 3a). The promoter, the rho-independent terminator and a putative upstream regulating sequence (URS) show extensive sequence similarities. The transcription start site was tentatively deduced from the consensus -10 element (T/G CTATACT) of RpoS-dependent promoters [31]. The presence of this RpoS promoter element is in agreement with our finding that RgsA sRNA was absent from an *rpoS* mutant of *P. aeruginosa* and that a reduced amount of this sRNA was present in an *rpoS* mutant of *P. fluorescens*(Figures 2a and 2b). In a database search, we did not find any sequence that is highly similar to the entire conserved URS sequence, suggesting that it might be a binding site for a specialized rather than for a global regulator. In particular, the URS motif is not related to the palindromic upstream activating sequence which is located upstream of the *rsmX*, *rsmY *and *rsmZ *genes and which is a potential GacA binding site [11,16]. We therefore suspect that the GacA effect on RgsA expression is likely to be indirect. The predicted size of RgsA (120 nt) agrees well with the length of the 1698 transcript observed in the initial Northern blots (Figure 1).

The genomic context of the *rgsA *gene in *P. fluorescens *Pf-5 and CHA0 is shown in Figure 3b. The flanking ORF PFL_1804 (coding for a regulator of the TetR family), which is located 800 bp upstream, is conserved only in *P. putida *KT2440. The downstream, divergently transcribed ORF PFL_1803 (a homolog of the *E. coli tatD *gene) is conserved in all *Pseudomonas *strains sequenced to date. In *E. coli*, the *tatD *product exhibits Mg-dependent DNase activity, but is not required for protein transport by the Tat pathway, contrary to what was originally expected [32].

### Regulation of *rgsA *gene expression in *P. aeruginosa *PAO1 and *P. fluorescens *CHA0

**Figure 4:**
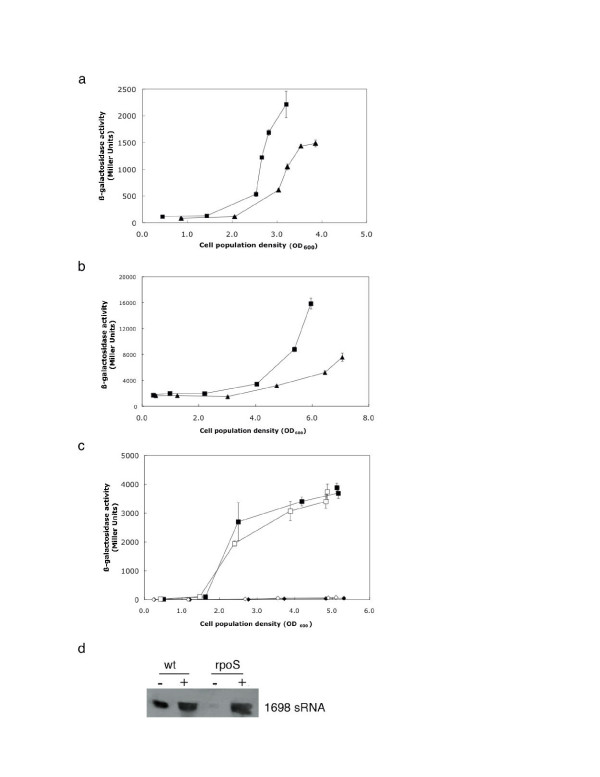
**Expression of *rgsA*-*lacZ *and *hcnA'-'lacZ *fusions**. **a. **β-Galactosidase activities of an *rgsA*_*PAO1*_-*lacZ *transcriptional fusion (pME7235) were determined in the *P. aeruginosa *wild-type PAO1 (squares) and in the *gacA *mutant PAO6281 (triangles). **b**. β-Galactosidase activities of an *rgsA*_*CHA0*_-*lacZ *transcriptional fusion (pME7234) were determined in the *P. fluorescens *wild-type CHA0 (squares) and in the *gacA *mutant CHA89 (triangles). **c. **β-Galactosidase activities of a chromosomal *hcnA*'-'*lacZ *translational fusion were determined in a *P. fluorescens *wild-type context (CHA207) carrying the empty pME6032 vector (filled squares) or the *rgsA *overexpressing plasmid pME7236 (open squares), and in the *rsmXYZ *triple mutant (CHA1145) carrying pME6032 (filled diamonds) or pME7236 (open diamonds). 1 mM IPTG was added when the cultures were inoculated. Each value in **a**, **b **and **c **is the average from three different cultures ± standard deviation. **d. **Verification of the overexpression of RgsA sRNA by Northern blot. Total RNA was purified from cultures of *P. fluorescens *CHA0 (wild type) and CHA815 (*rpoS *mutant) grown to stationary phase, carrying the empty pME6032 vector (-) or the *rgsA *overexpressing plasmid pME7236 (+).

To monitor the *rgsA *regulation by GacA during growth, we constructed *rgsA*_*PAO1*_-*lacZ *and *rgsA*_*CHA0*_-*lacZ *transcriptional fusions in plasmids pME7235 and pME7234, respectively. The primers used for these constructions (Figure 3b) carry a BamHI site, which enabled us to place the *lacZ *gene under the control of the respective *rgsA *promoter. At low cell population densities, the wild-type strains PAO1 and CHA0 as well as the *gacA *mutants PAO6281 and CHA89 exhibited similar basal levels of *lacZ *expression from the *rgsA-lacZ *constructs (Figures 4a and 4b). At high cell population densities, the *gacA *mutants showed an approximately two-fold reduction of β-galactosidase activity, compared with the activity in the wild-type strain. This finding is consistent with the Northern blot results (Figures 2a and 2b). The *rgsA*_*CHA0*_-*lacZ *fusion carried by pME7234 was also assayed in the *rpoS *mutant CHA815; at OD_600 _≈ 4.0, this fusion showed a 40% decrease of *lacZ *expression, compared with that in the wild-type CHA0 (data not shown). In conclusion, transcription of the *rgsA *gene is probably activated indirectly by GacA and directly by RpoS, which is assumed to bind to the -10 promoter element (Figure 3b), both in *P. aeruginosa *and *in P. fluorescens*.

### Role of RgsA sRNA in the Gac/Rsm regulatory cascade of *P. fluorescens *CHA0

**Figure 5:**
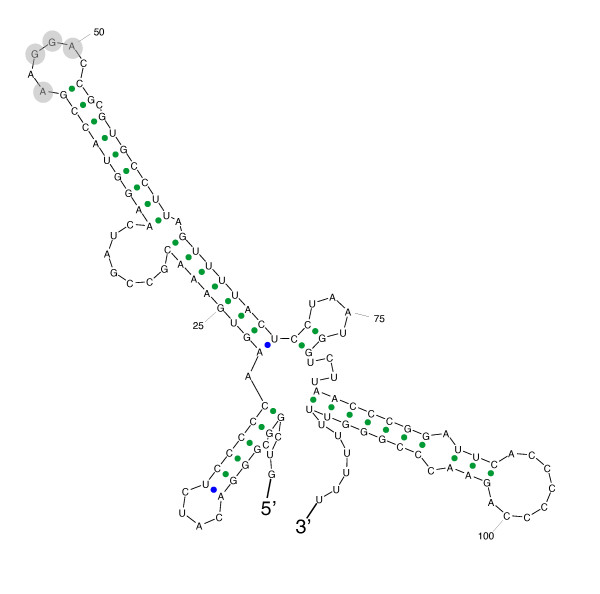
**Secondary structure of RgsA sRNA from *P. fluorescens *CHA0**. Secondary structure of RgsA as predicted by the SFold program [57]. A predicted RsmA-binding motif (ANGGA) at position 46–50 in an unpaired region is highlighted by grey circles.

A single, typical RsmA-binding motif (ANGGA) was found in an unpaired region of the RgsA sRNA of strain CHA0 (Figure 5). The GacA-controlled sRNAs RsmX, RsmY and RsmZ and their functional homologs each contain several GGA and extended ANGGA motifs [8]. In the case of RsmY, these motifs have been shown to be essential for sequestration of RsmA and its homolog RsmE in *P. fluorescens *[33]. We therefore investigated whether the RgsA sRNA might also have a titrating role, by testing whether *rgsA *overexpression would enhance the expression of a translational *hcnA*'-'*lacZ *fusion in *P. fluorescens *CHA0. Expression of *hcnA*, the first gene of the *hcn *operon involved in HCN biosynthesis, is tightly controlled by the Gac/Rsm cascade in strain CHA0. In an *rsmX rsmY rsmZ *triple mutant, artificial overexpression of *rsmX *rescues *hcnA*'-'*lacZ *expression [10]. We measured the expression of a chromosomal *hcnA*'-'*lacZ *fusion in the wild-type background and in the *rsmXYZ *triple mutant where the activity was almost totally lost (Figure 4c). When *rgsA*_*CHA0 *_was overexpressed in the wild-type strain (CHA207/pME7236), no significant difference was observed, compared with the native context (CHA207 containing the empty vector pME6032). Similarly, the *rgsA*_*CHA0 *_overexpressing plasmid pME7236 was unable to elevate *hcnA*'-'*lacZ *expression in the *rsmXYZ *triple mutant (CHA1145/pME7236), which was indistinguishable from the strain carrying the vector alone (CHA1145/pME6032) (Figure 4c). In a control experiment, we verified *rgsA*_*CHA0 *_overexpression by plasmid pME7236. Total RNA was extracted from strains carrying either pME7236 or the vector pME6032 and the amount of RgsA sRNA expressed in the cells was evaluated by Northern blotting. Overexpression of RgsA sRNA was detectable in the wild-type strain CHA0 and even more clearly in the *rpoS *mutant CHA815 (Figure 4d). In conclusion, the single RsmA-binding motif of RgsA was unable to derepress *hcnA *expression in *P. fluorescens*, confirming earlier evidence that multiple GGA motifs are required in sRNAs for effective sequestration of RsmA-like proteins [33].

The Gac/Rsm cascade modulates the response of *P. fluorescens *to stress imposed by hydrogen peroxide [14]. We constructed an *rgsA *deletion mutant of strain CHA0 and tested its survival after a 30-min exposure to 40 mM H_2_O_2 _in NYB with shaking. This experiment was carried out three times with triplicate cultures. Only 69 ± 16% of the mutant cells survived, compared with 94 ± 12% surviving wild type cells. (The initial viable count was set at 100%.) Thus, survival was significantly (*P *< 0.05) higher in the wild type than in the mutant. Overexpression of *rgsA *afforded no significant effect. These results suggest that RgsA may contribute to oxidative stress response.

## Discussion

The screening procedure adopted in our study enabled us to find evidence for 15 sRNAs in *P. aeruginosa *and other *Pseudomonas *spp. The identification of four of these sRNAs can be considered as a validation of the method, as they are either widespread in bacteria (4.5S RNA, RNase P RNA) or have been previously described in *P. aeruginosa *(PrrF1, PrrF2) [19]. Three additional sRNAs (491, 1698, 1887) confirm the existence of molecules that were previously identified by Livny *et al. *[20], and the observed lengths of these sRNAs are similar in both studies. Although our computational approaches are similar to those used by Livny *et al. *[20], we obtained a different output. On the one hand, we found evidence for eight new sRNAs. It is likely that by applying a 93% confidence threshold in the prediction of rho-independent terminators with TransTerm, we expanded the spectrum of intergenic regions considered to carry sRNA genes, whereas Livny *et al. *[20] had used a 96% threshold. On the other hand, 14 sRNAs detected by Livny *et al. *[20] were not identified in this study, either because the IgRs that produce them were discarded by our configuration of the QRNA program (this concerns sRNAs P1, P8, P9, P15, P18, P32, P34 and P35) or because we did not subject all 130 candidate IgRs to Northern blot analysis (this concerns our IgRs 967, 1023, 1554, 1633, 2429 and 2680, which correspond to P10, P11, P13, P14, P27 and P36, respectively). By introducing two additional genomes into the analysis, i.e., those of *P. fluorescens *Pf0-1 and *P. fluorescens *Pf-5, and by considering only the most widely conserved candidates we also demanded a higher level of conservation than did Livny *et al. *[20].

RsmY and RsmZ, the RsmA-binding sRNAs previously described in *P. aeruginosa *[16,34], were not revealed in our present study. The reasons for this may be that the RsmZ sRNAs of strains PAO1 and CHA0 have only 63% sequence identity (according to a needle global alignment; [35]), just below the 65% cut-off used. Although the RsmY sRNAs of strains PAO1 and CHA0 share 73% overall sequence identity in a needle alignment, the conserved segments of the RsmY sRNAs obtained with the BLASTn searches were short and shared an elevated sequence identity. In such cases the QRNA program, which needs sufficient substitutions that preserve secondary structures or codon bias, fails to identify and categorize the RNAs, explaining the absence of RsmY from our study.

In both *P. aeruginosa *PAO1 and *P. fluorescens *CHA0 the Gac/Rsm cascade regulates the expression of exoproducts such as HCN. In these organisms, a double *rsmY rsmZ *and triple *rsmX rsmY rsmZ *mutant, respectively, have the same exoproduct phenotype as *gacA *mutants [10,16]. However, in strain CHA0, GacA has an additional function as a regulator of *rpoS *expression and, consequently, of the response to oxidative stress [14]. This raised the question of whether there might be further GacA-controlled sRNAs, which might be involved in stress response. This idea motivated us to test the sRNAs listed in Table 1 for regulation by GacA. In both strains PAO1 and CHA0, the RgsA (1698) sRNA was found to have reduced expression in *gacA *mutants, by comparison with the wild-type strains (Figure 2). Moreover, RpoS was needed for *rgsA *induction in both strains during stationary phase (Figure 2). We confirmed this regulation in both *P. aeruginosa *and *P. fluorescens *by measuring the expression of *rgsA*-*lacZ *fusion constructs (Figures 4a and 4b). We also found that the overexpressed RgsA sRNA was unable to relieve translational repression of the *hcnA *gene in *P. fluorescens *(Figure 4c), which is consistent with the fact that this sRNA has only a single GGA motif. Previously, it was shown that an RsmY mutant having a single GGA motif has lost the ability to bind the RsmA and RsmE proteins [33].

In response to H_2_O_2 _stress, a *P. fluorescens *mutant deleted for the *rgsA *gene showed a reduced ability to survive, compared with the wild type. The mechanism by which this protective effect of RgsA occurs remains to be discovered.

## Conclusion

In this study we have found evidence for eight new sRNAs in *P. aeruginosa*. This brings the total of detected sRNAs to almost 30 in this organism. For the vast majority of them, the physiological roles are unknown. We discovered that the RgsA sRNA, which was termed P16 by Livny *et al. *[20], is transcribed from an RpoS-dependent promoter under positive, probably indirect control of GacA in *P. aeruginosa *PAO1 as well as in *P. fluorescens *CHA0. In the latter organism, the RgsA sRNA appears to be unable to sequester the RsmA and RsmE proteins and is unlikely to have a role in the regulation of exoproduct formation, but helps protect the bacterium from H_2_O_2_.

## Methods

### Bacterial strains and growth conditions

We used *P. aeruginosa *PAO1 (ATCC 15692) and its derivatives PAO1-rpoS [30] and PAO6281 (*gacA*::Ω-Sm/Sp) [13], *P. fluorescens *CHA0 [36], CHA89 (*gacA*::Km^r^) [37], CHA207 (*hcnA*'-'*lacZ*) [38], CHA815 (Δ*rpoS*) [9], CHA1145 (*rsmXYZ*, *hcnA*'-'*lacZ*) [10] and CHA1181 (Δ*rgsA*, this study). *E. coli *DH5α [39] served for gene cloning. Strains were routinely grown in nutrient yeast broth (NYB; 2.5% [wt/vol] nutrient broth, 0.5% [wt/vol] yeast extract) with shaking, or on nutrient agar (4% [wt/vol] blood agar base, 0.5% [wt/vol] yeast extract). When required, ampicillin at 100 μg ml^-1 ^or tetracycline at 25 μg ml^-1 ^(100 μg ml^-1 ^for pseudomonads) were added. For some RNA extractions, strains were also grown in GGP medium [40] or in MME minimal medium [41]. To monitor β-galactosidase expression qualitatively, 5-bromo-4-chloro-3-indolyl-β-D-galactoside (Xgal) was incorporated into solid media at a final concentration of 0.02%. Routine incubation temperatures were 37°C for *E. coli *and *P. aeruginosa *and 30°C for *P. fluorescens*. Survival of stationary phase *P. fluorescens *was assayed in NYB following H_2_O_2 _stress as described previously [14]. Statistical significance was assessed by Student's t-test.

### DNA manipulations and cloning procedures

DNA manipulations were carried out according to standard procedures [39]. Plasmid isolation was performed using the cetyl-trimethyl-ammonium bromide method (CTAB) [42] for small preparations, and the JetStar kit (Genomed GmbH) for large preparations. DNA fragments were purified from agarose gels with the MinElute Gel Extraction Kit or the Qiaquick Gel Extraction Kit (Qiagen Inc.). Transformation of the strains was done by electroporation [43]. DNA sequencing was carried out by Microsynth (Microsynth AG). Polymerase chain reaction was carried out as previously described [11].

### Plasmid constructions

To generate pME7234 carrying a transcriptional *lacZ *fusion to the *rgsA *gene from *P. fluorescens *CHA0, a 510-bp fragment containing the *rgsA *sRNA promoter was amplified from chromosomal DNA by PCR using primers 1698Fus1 (EcoRI) and 1698Fus2 (BamHI) listed in Additional file 3, digested by EcoRI and BamHI, and inserted into EcoRI/BamHI-cut pBLS (pBluescript II KS+ cloning vector, ColE1 replicon, Ap^R^, Stratagene). After sequencing, the fragment was cloned into the shuttle vector pME6016 [44]. The resulting plasmid pME7234 carries the *rgsA*_*CHA0 *_promoter controlling the expression of the *lacZ *gene. Similarly, we constructed pME7235 carrying a transcriptional *lacZ *fusion to the *rgsA *gene from *P. aeruginosa *PAO1 using primers 1698Fus3 (EcoRI) and 1698Fus4 (BamHI) (Additional file 3) and analogous cloning steps.

To overexpress *rgsA *from *P. fluorescens *CHA0, plasmid pME7236 was constructed in several steps. First, a 180-bp fragment containing the *rgsA *gene was amplified from chromosomal DNA using the primers 1698Sur4 (EcoRI, PstI) and 1698Sur2 (HindIII) (Additional file 3), digested with EcoRI and HindIII, and inserted into EcoRI/HindIII-cut pBLS. A 200-bp fragment was then excised from pBLS with EcoRI and KpnI and cloned into pME6032 [9]. The Shine-Dalgarno sequence carried by pME6032 was removed, as it is unsuitable for the expression of a non-coding sRNA gene, by a second round of PCR using the primers 6032K (HpaI) and 1698Sur6 (PstI) (Additional file 3), digestion by HpaI and PstI, and cloning. The resulting construct pME7236 carries the pME6032 *tac *promoter 13 nt upstream of the *rgsA *+1 site. The introduction of the PstI restriction site downstream the *tac *promoter added 3 nt and modified 2 nt of the RgsA sRNA at the 5' end (5'-TCTGCAGGCGGG... instead of 5'-GTCGGCGGG...).

### Construction of an *rgsA *mutant of *P. fluorescens*

A deletion of the *rgsA *gene in the *P. fluorescens *CHA0 chromosome was made as follows. A 860-bp fragment containing the upstream region and the *rgsA *promoter was amplified by PCR with primers 1698DelE (EcoRI) and 1698Del3 (NcoI) (Additional file 3). A 750-bp fragment containing the *rgsA *terminator and most of the downstream PFL_1803 gene was amplified by PCR with primers 1698Del4 (NcoI) and 1698DelX (XbaI) (Additional file 3). Both fragments were digested with NcoI and ligated together; in the resulting ~1.6-kb fragment there remained 11 bp between the transcription start site and the terminator of the *rgsA *gene. The fragment was digested with EcoRI and XbaI and cloned into pME3087 (suicide vector; ColE1 replicon; Tc^r^; [36]). *E. coli *DH5α was transformed with the resulting pME3087 derivative and used in a triparental mating with the helper HB101/pME497 [45]. In this way the deleted *rgsA *gene (Δ*rgsA*) was introduced into CHA0, giving CHA1181.

### Computational search parameters in the screening for sRNAs

Our screening was limited to the IgRs of the *P. aeruginosa *PAO1 genome having a length exceeding 50 bp. The corresponding 3168 sequences were listed in FASTA format and compared by BLASTn to the genome sequences of *P. putida *KT2440 [46], *P*. *syringae *pv. *tomato *DC3000 [47], *P*. *fluorescens *SBW25 [48], *P*. *fluorescens *Pf0-1) [49], and *P*. *fluorescens *Pf-5 [50]. We fixed a cut-off at 65% sequence identity. The PAO1 sequences fulfilling this criterion, together with the homologous sequences of at least another *Pseudomonas *strain, were given as input data into the program QRNA [5]. Briefly, this program is able to compare each of the PAO1 sequences with a paired sequence, and tests the pattern of substitutions observed in the pairwise alignment of two homologous sequences. Three models of substitution are analyzed. Model (i) assumes that mutations occur in a position-independent manner (called OTH); model (ii) assumes that in homologous coding regions mutations often result in conservative amino acid substitutions (called COD); model (iii) assumes that the pattern of mutations significantly conserves the secondary structure in a homologous RNA (called RNA). For each alignment QRNA establishes scores according to each model and calculates to which category the aligned sequences most probably belong. For IgRs, sequences can be described as COD if after the alignment with their homologous sequences they do not have enough substitutions reflecting secondary structure conservation and present a (probably fortuitous) codon usage bias. Using QRNA we obtained a list of 162 intergenic regions that share coding (COD) or structural homology (RNA) with at least one related genome sequence (see Additional file 1).

### Sequence analysis tools

FUZZNUC, a free access nucleic acid pattern search program [35], was used to search for conserved motifs in IgRs, e.g. for RpoD (σ^70^) binding sites (TTGACAN_(17)_TATAAT), RpoN (σ^54^) binding sites (TGGCACN_(5)_TTGCW, where W is A or T, based on Barrios *et al. *[51]) or for the -10 element of RpoS (σ^38^)-binding sites (TGN_(0–2)_CCATACT, according to Lacour *et al. *[52]). We used CLUSTALW [53] for multiple sequence alignments and TransTerm [24] to identify rho-independent terminators in IgRs. The analysis was done using the entire PAO1 chromosomal sequence as the template and the list of PAO1 ORFs (such that the program can distinguish the intragenic from the intergenic parts of the chromosomal sequence); both were obtained from the *P. aeruginosa *PAO1 sequencing project site [54]. The confidence threshold was set to 93%.

### RNA extraction and Northern blots

RNAs used for Northern blot analysis were isolated using a hot acid phenol extraction protocol, based on the technique described by Massé* et al. *[55]. The original protocol was modified as follows: 5 ml of cell culture was centrifuged and resuspended in 5 ml of TKM buffer (10 mM Tris-HCl, 10 mM KCl, 5 mM MgCl_2_, pH 7.5), before mixing with lysis buffer (320 mM Na acetate, 8% [wt/vol] Na dodecylsulfate, 16 mM EDTA, pH 4.6, treated with di-ethyl-pyrocarbonate [DEPC] and autoclaved). DIG-labelled probes were obtained as follows: PCR fragments were synthesized with *P. aeruginosa *or *P. fluorescens *genomic DNA as the template and pairs of primers listed in the Additional file 3. For each probe, the first primer was designed to hybridize to the proximal part of the upstream ORF (forward primer, indicated by F) and the second to hybridize to the proximal part of the downstream ORF (reverse primer, indicated by R). The fragments obtained corresponded to the IgRs to be analyzed and were then used as templates for a second PCR, which was carried out with the same pairs of primers as before, but in the presence of DIG DNA labeling mix (DNA labeling Mix, 10 × conc., Roche). RNAs (~5 μg per lane) were separated on a denaturing urea-polyacrylamide gel and analyzed by Northern blotting as previously described [11], with minor modifications: RNAs were transferred by electroblotting (20 min at 150 mA) onto a charged Nylon membrane (Hybond-N^+^, GE-Amersham) and revealed by hybridization with the DIG-labeled probes described above and exposure to a light-sensitive film (Super RX, Fujifilm). 5S rRNA served as loading control. For this purpose, a 5S-rDNA probe was synthesized with primers 5S-rRNA-1 and 5S-rRNA-2 (Additional file 3) and DIG-labeled. The membranes could be used a second time for Northern blot detection of 5S rRNA. To completely detach the probe previously hybridized, membranes were rinsed with DEPC-treated H_2_O and immersed twice in 0.2 M NaOH containing 0.1% Na dodecylsulfate for 15 min. The membranes were then rinsed with 2× SSC solution [39] and used in further Northern blots.

### β-Galactosidase assays

*P. fluorescens *cells were grown in 20 ml of NYB, in 100 ml Erlenmeyer flasks. Triton X-100 (0.1% wt/vol) was added to the cultures to avoid cell aggregation. Samples were taken during various growth phases and permeabilized with 5% toluene. β-Galactosidase activities were then measured according to the Miller method [56].

## Abbreviations

BLAST, Basic Local Alignment Search Tool; CTAB, cetyl-trimethyl-ammonium bromide; DEPC, di-ethyl-pyrocarbonate; DIG, digoxigenin; EDTA, ethylene-di-amine-tetraacetic acid; GGP, iron-limited medium; HCN, hydrogen cyanide; IgR, intergenic region; MME, minimal medium E; NYB, nutrient yeast broth; SRNA, small RNA; URS, upstream regulating sequence; Xgal, 5-bromo-4-chloro-3-indolyl-β-D-galactoside;

## Authors' contributions

NG designed and carried out experiments, and drafted manuscript; SH and TJ executed the bioinformatics search; CV, EK and CR performed Northern blots; DH directed research and wrote the manuscript.

## Supplementary Material

Additional file 1 **Intergenic regions**. List of IgRs of *Pseudomonas aeruginosa *PAO1 giving a positive result in QRNA analysis. Click here for file

Additional file 2 **Predicted coordinates of three sRNA genes**. Figure showing the predicted coordinates of sRNA genes in IgRs 645, 1887 and 2315. Click here for file

Additional file 3 **Primers**. Primers used in this study for Northern blots and recombinant DNA work. Click here for file
