# Supplementary material for: Genome-wide search reveals a novel GacA-regulated small RNA in Pseudomonas species
Source: BMC Genomics. 2008 Apr 13;9:167. doi: 10.1186/1471-2164-9-167 (PMC2375449; doi:10.1186/1471-2164-9-167)
Supplement: Additional file 2 — Predicted coordinates of three sRNA genes. Figure showing the predicted coordinates of sRNA genes in IgRs 645, 1887 and 2315. [file 1471-2164-9-167-S2.pdf]

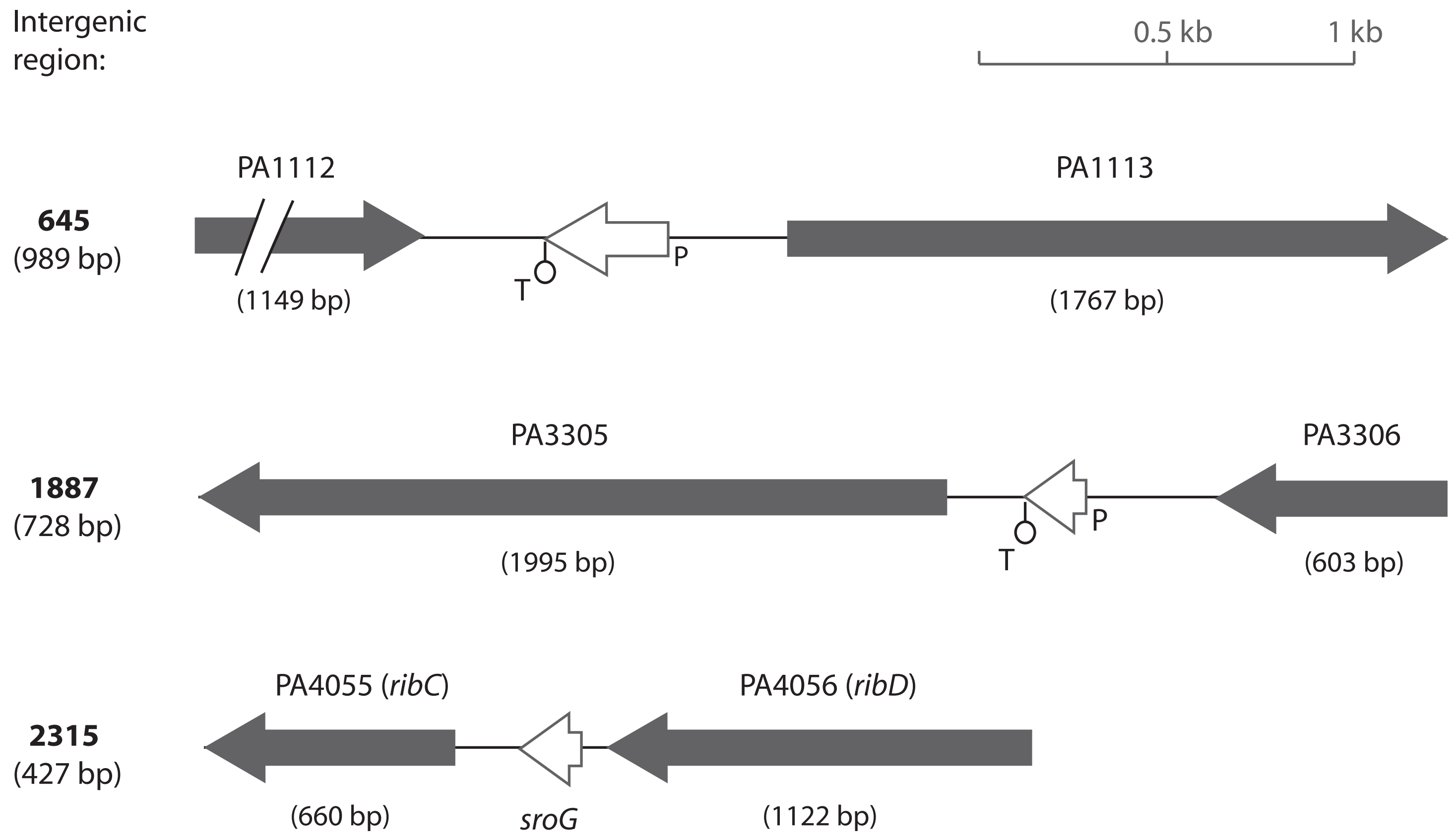

Additional file 2: Predicted coordinates of three sRNA genes (645, 1887, 2315). These predictions are based on an analysis of putative RpoD-dependent promoters (P), putative ρ-independent terminators (T) or sequence homology (in case of *sroG*; Vogel *et al.*, 2003). The coordinates predicted are: 1205239 - 1205035 (IgR 645); 3705522 - 3705315 (IgR 1887); 4537006 - 4536845 (IgR 2315).
